# Calaxin is required for cilia-driven determination of vertebrate laterality

**DOI:** 10.1038/s42003-019-0462-y

**Published:** 2019-06-20

**Authors:** Keita Sasaki, Kogiku Shiba, Akihiro Nakamura, Natsuko Kawano, Yuhkoh Satouh, Hiroshi Yamaguchi, Motohiro Morikawa, Daisuke Shibata, Ryuji Yanase, Kei Jokura, Mami Nomura, Mami Miyado, Shuji Takada, Hironori Ueno, Shigenori Nonaka, Tadashi Baba, Masahito Ikawa, Masahide Kikkawa, Kenji Miyado, Kazuo Inaba

**Affiliations:** 1 Shimoda Marine Research Center, University of Tsukuba, Shimoda, 415-0025 Japan; 2 Department of Reproductive Biology, National Center for Child Health and Development, Tokyo, 157-8535 Japan; 3 Department of Life Science, School of Agriculture, Meiji University, Kanagawa, 214-8574 Japan; 4 Research Institute for Microbial Diseases, Osaka University, Osaka, 565-0871 Japan; 5 Department of Cell Biology and Anatomy, Graduate School of Medicine, The University of Tokyo, Tokyo, 113-0033 Japan; 6 Department of Molecular Endocrinology, National Research Institute for Child Health and Development, Tokyo, 157-8535 Japan; 7 Department of Systems BioMedicine, National Research Institute for Child Health and Development, Tokyo, 157-8535 Japan; 8 Molecular Function & Life Sciences, Aichi University of Education, Aichi, 448-8542 Japan; 9 Spatiotemporal Regulations Group, Exploratory Research Center on Life and Living Systems (ExCELLS), Okazaki, 444-8585 Japan; 10 Laboratory for Spatiotemporal Regulations, National Institute for Basic Biology, Okazaki, 444-8585 Japan; 11 Faculty of Life and Environmental Sciences, and Life Science Center for Survival Dynamics Tsukuba Advanced Research Alliance (TARA), University of Tsukuba, Tsukuba, 305-8577 Japan

**Keywords:** Dynein, Ciliogenesis

## Abstract

Calaxin is a Ca^2+^-binding dynein-associated protein that regulates flagellar and ciliary movement. In ascidians, calaxin plays essential roles in chemotaxis of sperm. However, nothing has been known for the function of calaxin in vertebrates. Here we show that the mice with a null mutation in *Efcab1*, which encodes calaxin, display typical phenotypes of primary ciliary dyskinesia, including hydrocephalus, *situs inversus*, and abnormal motility of trachea cilia and sperm flagella. Strikingly, both males and females are viable and fertile, indicating that calaxin is not essential for fertilization in mice. The 9 + 2 axonemal structures of epithelial multicilia and sperm flagella are normal, but the formation of 9 + 0 nodal cilia is significantly disrupted. Knockout of calaxin in zebrafish also causes *situs inversus* due to the irregular ciliary beating of Kupffer’s vesicle cilia, although the 9 + 2 axonemal structure appears to remain normal.

## Abstract (web summary)

Keita Sasaki, Kogiku Shiba, Akihiro Nakamura et al. show that mice lacking calaxin have intact fertility despite showing primary cilia dyskinesia and abnormal 9 + 0 nodal cilia formation, while loss of calaxin in zebrafish causes *situs inversus* but does not affect 9 + 2 axonemal formation. Together, these findings suggest a specific role for calaxin in 9 + 0 nodal cilia development.

## Introduction

Motile cilia and flagella are organelles that have been conserved through evolution^1–3^. They possess internal cytoskeletal structures, axonemes, that are composed of nine outer doublet microtubules and two central singlet microtubules (9 + 2 structure)^4,5^. Two types of projection extend from each microtubule doublet, the outer and inner dynein arm (ODA and IDA), both of which are large, multi-subunit complexes consisting of heavy, intermediate, and light chains. Dynein heavy chains (HCs) are motor subunits that hydrolyze ATP to convert chemical energy into mechanical energy for microtubule movement. The intermediate and light chains (ICs and LCs) assemble and regulate the motor subunits.

Genetic defects of the dynein components cause primary ciliary dyskinesia (PCD), a human ciliopathy disease^6–8^. PCD is characterized by defects in the motility of cilia and flagella in a variety of cells, including sperm, and in tissues of the trachea, ependyma, and embryonic node. By utilizing both mice and zebrafish as model systems, it is possible to acquire important insights into the phenotypes and mechanism of PCD^9–11^.

PCD is most often caused by defects in a subunit of the ODA, including HCs (DNAH5 and DNAH11), ICs (DNAI1, DNAI2 and TXNDC3/NME8), LCs (DNAL1 and TECTE3), components of the docking complex (CCDC114, TTC25), and ODA-associated proteins (CCDC151, CCDC103, and ARMC4)^12–22^. These mutations result in the complete or partial absence of the ODA, leading to PCD^23^. An exception to this is patients with mutations in *DNAH11* (previously termed, *Left-Right Dynein*, *LRD*)^24^. Like PCD patients with mutations in other ODA-coding genes, those with *DNAH11* mutations have respiratory defects, situs abnormalities and are infertile. However, their ciliary structures are normal with normal ODAs^24,25^.

**Fig. 1:**
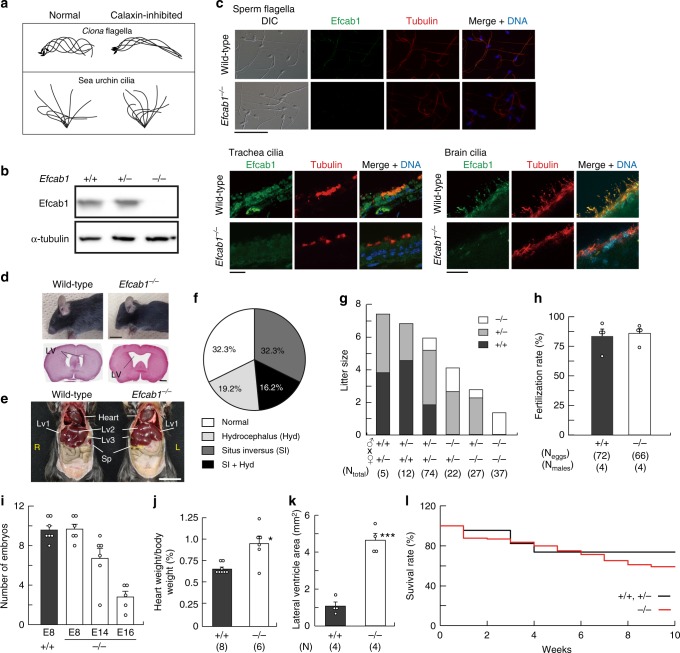
Generation and phenotypes of *Efcab1* mutant mice. **a** Function of calaxin in the propagation of flagellar and ciliary waveforms. Typical waveforms of *Ciona* sperm flagella and sea urchin embryonic cilia in normal and calaxin-inhibited conditions are shown. Modified from previous publications^30,32^. **b** Immunoblot analysis of *Efcab1*^−/−^ sperm by anti-Efcab1 and anti-acetylated-α-tubulin antibodies. **c** Immunofluorescence analysis of *Efcab1*^−/−^ sperm flagella, trachea and ependymal cilia by anti-Efcab1 and anti-acetylated-α-tubulin antibodies. Scale bar, 50 μm (top) and 25 μm (bottom left and right). **d** *Efcab1*^−/−^ mouse showing hydrocephalus (upper; head morphology, scale bar = 1 cm, lower; 5 µm paraffin section stained with hematoxylin-eosin) and **e** *situs inversus*. LV, lateral ventricle; Lv, liver; Sp, spleen. **f** Percentage of *Efcab1*^−/−^ mice with hydrocephalus and/or *situs inversus*. *N* = 130 (71; male, 59; female). **g** Average litter size from parents of different genotypes. **h** The rate of in vitro fertilization of eggs with epididymal sperm collected from *Efcab1*^+/+^ and *Efcab1*^−/−^ mice. Numbers in parentheses indicate the number of eggs examined from triplicate experiments. **i** Number of embryos at E8, E14 and E16 after mating with *Efcab1*^+/+^ or *Efcab1*^−/−^ male mice. **j** The ratio between heart and body weight in *Efcab1*^+/+^ and *Efcab1*^−/−^ mice. **k** Comparison of lateral ventricle area between *Efcab1*^+/+^ and *Efcab1*^−/−^ mice. **l** Survival rate at 10 weeks. *N* = 46 (*Efcab1*^+/+^ and *Efcab1*^+/−^) and *N* = 198 (*Efcab1*^−/−^). Values indicate mean ± SEM. *p < 0.05, ***p < 0.001 vs. *Efcab1*^+/+^ (Student’s *t*-test)

The motility of cilia and flagella is modulated in response to several extracellular stimuli^26–28^. The most critical intracellular factor mediating these changes is Ca^2+^. Calaxin is a neuronal calcium sensor protein first described in the sperm of the ascidian *Ciona intestinalis*^29–31^. It directly binds to the β-type heavy chain (orthologous to *Chlamydomonas γ* heavy chain^3^) of the ODA in a Ca^2+^-dependent manner and regulates the propagation of the asymmetric flagellar wave. It is also necessary for changes in swimming direction during sperm chemotaxis^30^ (Fig. 1a). In sea urchin embryos, calaxin is a critical regulator for the coordinated movements of monocilia and is a prerequisite for the establishment of ciliary orientation^32^ (Fig. 1a), which is generally thought to be determined by the planar cell polarity. Calaxin is a Ca^2+^ sensor that has evolved in the opisthokont (animal + fungi) lineage^3^; however, it has not been widely studied, particularly in vertebrates.

The initial aim of this study was to elucidate the role of calaxin in vertebrate male fertility, particularly how sperm chemotaxis contributes to the success of vertebrate internal fertilization. We generated knockout mice lacking the gene encoding calaxin, *Efcab1*. Both male and female *Efcab1*^−/−^ mice were unexpectedly fertile. However, many *Efcab1*^−/−^ mice showed hydrocephalus and visceral inversion, both of which are typical features of ciliopathy, without apparent changes in 9 + 2 axonemal structures. Intriguingly, calaxin knockout caused a drastic loss of nodal cilia, whereas other cilia and flagella were normally formed.

## Results

### Calaxin knockout mice exhibit PCD but are fertile

To elucidate the physiological function of calaxin in vertebrates, we generated a knockout mouse in which exon 4 of *Efcab1*, the gene encoding calaxin, was genetically disrupted by homologous recombination (Supplementary Fig. 1a–c). No calaxin expression was observed in the sperm, trachea or ependyma of *Efcab1*^−/−^ mice either at mRNA (Supplementary Fig. 1c) or at protein level (Fig. 1b, c, Supplementary Fig. 1d). Notable phenotypes of postnatal *Efcab1*^−/−^ mice were hydrocephalus and *situs inversus* in 35% and 49% of offspring, respectively (Fig. 1d, e), with both phenotypes present in 16% of offspring (Fig. 1f).

In *Ciona*, calaxin is essential for chemotaxis of the sperm to the egg; however, both male and female *Efcab1*^−/−^ mice were fertile, although litter sizes when either or both parents were *Efcab1*^−/−^ were significantly lower compared with litters from wild-type parents (Fig. 1g). The litter size from *Efcab1*^−/−^ males and females was 1~2, whereas that from wild-type mice was 7~8. However, *Efcab1*^−/−^ sperm showed the same in vitro fertilization rate as wild-type sperm (Fig. 1h). The number of *Efcab1*^−/−^ embryos was almost the same as that of wild-type embryos at embryonic day 8 (E8) but after E14 the number of *Efcab1*^−/−^ embryos declined (Fig. 1i). *Efcab1*^−/−^ male and female offspring were born at non-Mendelian frequency with a deficit of *Efcab1*^−/−^ mice (Supplementary Table 1), indicating homozygotic embryonic lethality. *Efcab1*^−/−^ mice showed both cardiac hypertrophy (Fig. 1j) and enlargement of brain ventricles (Fig. 1d, k). Hydrocephalus emerges after birth and is not always lethal^33^; therefore, the cause of embryonic lethality is most likely to result from cardiac defects that often accompany PCD^34^. Surviving *Efcab1*^−/−^ mice showed a similar survival rate to wild-type mice (more than 10 weeks) (Fig. 1l). A bacterial artificial chromosome containing the entire *Efcab1* gene (Supplementary Fig. 2a, b) rescued the expression of calaxin protein (Supplementary Fig. 2c) and the PCD phenotypes of *Efcab1*^−/−^ mice (Supplementary Fig. 2d), clearly indicating the distinct roles of calaxin in ciliary function.

### Cilia of *Efcab1*^−/−^ mice have morphologically normal 9 + 2 axonemes

**Fig. 2:**
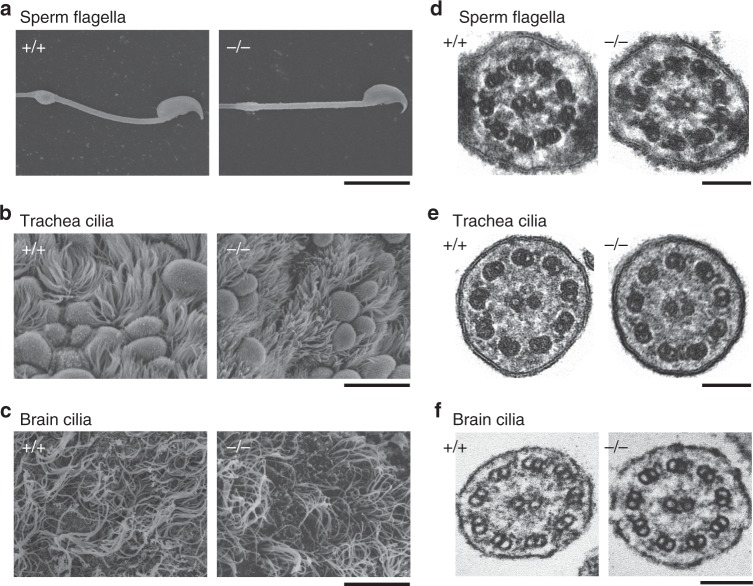
Ultrastructures of sperm flagella, trachea cilia and brain cilia in *Efcab1*^−/−^ mice. Sperm flagella (**a**, **d**), trachea cilia (**b**, **e**) and brain ependymal cilia (**c**, **f**) from *Efcab1*^+/+^ and *Efcab1*^−/−^ mice are shown. Images are from scanning electron microscopy (**a**–**c**) and thin-section electron microscopy (**d**–**f**). Scale bars: 10 µm (**a**–**c**), 100 nm (**d**–**f**)

Normal sperm flagella were observed by differential interference contrast (DIC) light microscopy (Fig. 1c) and scanning electron microscopy (Fig. 2a) in *Efcab1*^−/−^ male mice (Figs. 1c, 2a); similarly no abnormality was observed in the multicilia of tracheal or ependymal epithelia by scanning electron microscopy (Fig. 2b, c). Transmission electron microscopy of sperm flagella, trachea cilia and ependymal cilia revealed that the ODAs were mostly intact in *Efcab1*^−/−^ mice (Fig. 2d–f); in rare cases a few ODAs were absent from some doublet microtubules (Supplementary Fig. 3).

### *Efcab1*^−/−^ flagella and cilia show reduced motility and fluid flows

**Fig. 3:**
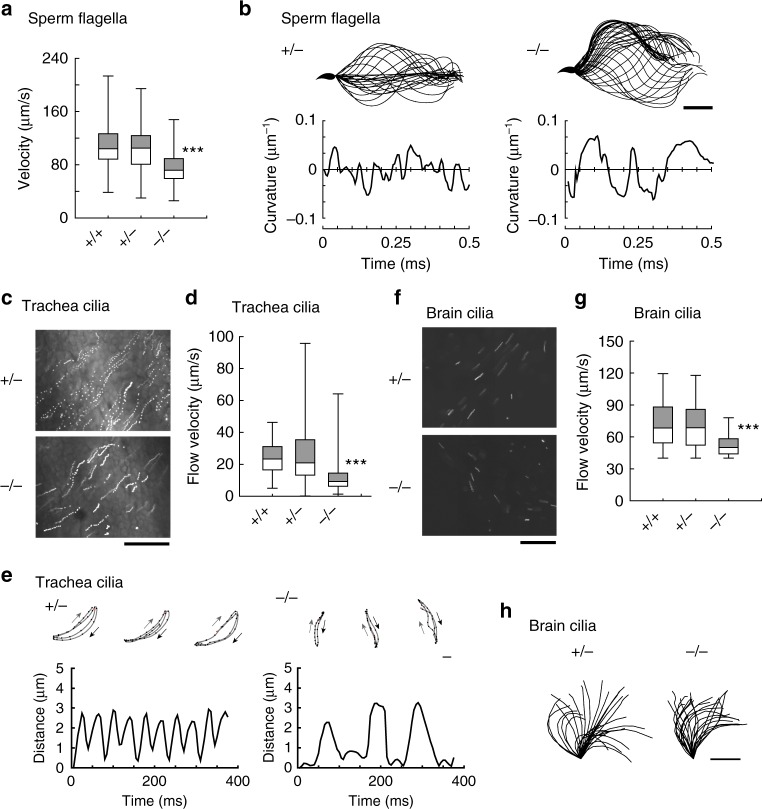
Motility of sperm flagella and epithelial cilia in *Efcab1*^−/−^ mice. **a** Average sperm curvilinear velocity. **b** Flagellar bending patterns (upper) and curvatures (lower) of sperm in *Efcab1*^+/−^ and *Efcab1*^−/−^ mice. Images at 2 ms intervals are overwritten (25 or 43 images for *Efcab1*^+/−^ and *Efcab1*^−/−^ sperm, respectively). Flagellar curvatures at 50 μm from the base are plotted against time. **c** Trajectories of fluorescent beads in trachea of *Efcab1*^+/−^ and *Efcab1*^−/−^ mice. Fifteen images acquired at 0.2 s intervals are superimposed. **d** Flow velocity of fluorescent beads in trachea cilia. **e** Movement of trachea cilia in *Efcab1*^+/−^ and *Efcab1*^−/−^ mice. Trajectories of fluorescent beads bound to ciliary tips (upper) are shown with effective (black) and recovery (gray) stroke paths, with changes in distance of bound fluorescent bead (lower). **f** Movement of ependymal cilia. Trajectories of fluorescent beads are shown. Fifty images acquired at 5 ms intervals are superimposed. **g** Flow velocity of fluorescent beads in ependymal cilia. **h** Ciliary bending patterns in *Efcab1*^+/−^ and *Efcab1*^−/−^ mice. Images at 2 ms intervals are overwritten (30 images for *Efcab1*^+/−^ and 25 images for *Efcab1*^−/−^ nodal cilia). Scale bar: 20 µm (**b**), 50 µm (**c**), 50 µm (**c**, **f**), 10 µm (**h**). Boxes correspond to the first and third quartiles, and whiskers extend to the full range of the data. ***p < 0.001 vs. *Efcab1*^+/+^ (Student’s *t*-test)

*Efcab1*^−/−^ mice were fertile, with *Efcab1*^−/−^ sperm showing the same degree of in vitro fertility as wild-type sperm (Fig. 1h), indicating that calaxin and its regulation of sperm motility are not essential for successful fertilization. To examine the detailed role of calaxin in the regulation of flagellar motility, we analyzed the flagellar waveform and its propagation in *Efcab1*^−/−^ sperm. The swimming velocities of *Efcab1*^+/+^ and *Efcab1*^+/−^ sperm were 109.2 ± 16.4 and 103.1 ± 15.7 µm/s, whereas that of *Efcab1*^−/−^ sperm was significantly decreased (74.9 ± 12.5 µm/s) (Fig. 3a). This decrease most likely resulted from abnormal wave propagation; *Efcab1*^−/−^ sperm showed transient arrest of pro-hook bend propagation (Fig. 3b; Supplementary Movies 1 and 2). Nonetheless, *Efcab1*^−/−^ sperm were fertile.

Tracheal cilia of *Efcab1*^−/−^ mice also exhibited active motility (Supplementary Movies 3 and 4). However, analysis of fluid flow in trachea using fluorescent beads revealed that the flow velocity in *Efcab1*^−/−^ mice was decreased to almost half that of wild-type mice (Fig. 3c, d; Supplementary Movies 5 and 6). High-speed filming of the trajectories of fluorescent beads attached to the tips of cilia showed that the frequency of ciliary beating was significantly decreased in *Efcab1*^−/−^ mice (Fig. 3e; Supplementary Movies 7 and 8). The velocity of the recovery stroke in *Efcab1*^−/−^ cilia was drastically slower than that in *Efcab1*^+/−^ cilia.

As with the case of trachea cilia, ependymal cilia of *Efcab1*^−/−^ mice also showed active motility (Fig. 3f; Supplementary Movies 9 and 10). Analysis of the fluid flow being driven by ependymal cilia showed that the flow velocity was slightly decreased in *Efcab1*^−/−^ mice compared with that in wild-type cilia (Fig. 3g; Supplementary Movie 11 and 12). This was most likely due to the cilia having a narrower range of effective stroke (Fig. 3h), as observed in the calaxin-knockdown embryos of sea urchins^32^.

### Calaxin knockout causes a distinct defect in nodal cilia formation

**Fig. 4:**
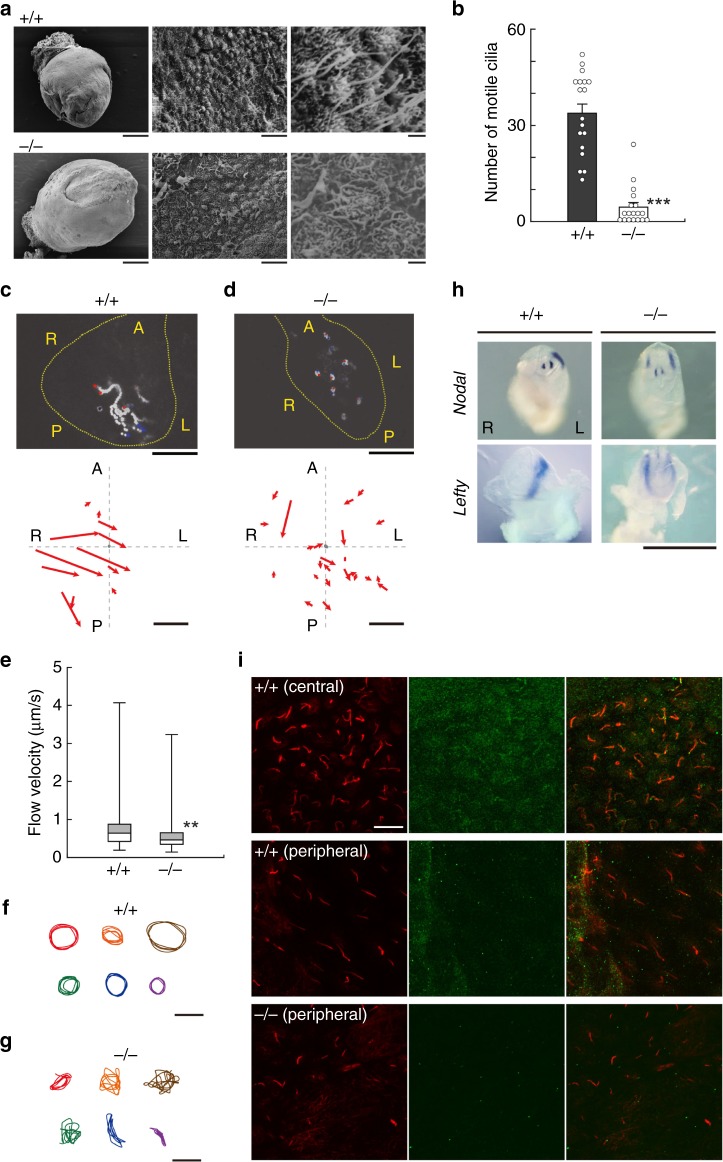
Appearance and motility of nodal cilia in *Efcab1*^−/−^ mice. **a** Scanning electron microscopy of E7.5 embryo and nodal cilia in *Efcab1*^+/+^ and *Efcab1*^−/−^ mice. Left, whole embryo; middle, node area; right, magnified image of nodal cilia. **b** Number of motile cilia in the node. *N* (embryos) = 18 (*Efcab1*^+/+^) and *N* = 18 (*Efcab1*^−/−^). **c**, **d** Nodal flow in *Efcab1*^+/+^ (**c**) or *Efcab1*^−/−^ (**d**) E7.5 embryos. Upper; Trajectories of fluorescent beads driven by nodal flow. Sixty images acquired at 0.25 s intervals are superimposed. Red and blue dots indicate the start and end points of the superimposition, respectively. Lower; direction and distance traveled by fluorescent beads in *Efcab1*^+/+^ or *Efcab1*^−/−^ nodal flows. Arrow length represents bead path and distance for 5 s. A, anterior; P, posterior; R, right; L, left. **e** Flow velocity of fluorescent beads. Boxes correspond to the first and third quartiles, and whiskers extend to the full range of the data. *N* (beads) = 112 from four embryos (*Efcab1*^+/+^) and *N* = 46 from 10 embryos (*Efcab1*^−/−^). **f**, **g** Trajectories of fluorescent beads binding to the tip of nodal cilia in *Efcab1*^+/+^ (**f**) and *Efcab1*^−/−^ (**g**) mice. **h** Whole-mount in situ hybridization analysis of *Nodal* and *Lefty* expression in E8.0 embryos. R, right; L, left. Scale bars: 200 µm (**a**, left), 10 µm (**a**, middle, **c**, **d**), 1 µm (**a**, right), 4 µm (**f**, **g**), 1 mm (**h**). **i** Immunofluorescent localization of Efcab1 in the central and peripheral regions of node in *Efcab1*^*+/+*^ and *Efcab1*^−/−^ mice. Images with anti-Efcab1 (green) and anti-acetylated-α-tubulin (red) antibodies are shown. Scale bars: 10 µm. **p < 0.01, ***p < 0.001 vs. *Efcab1*^+/+^ (Student’s *t*-test)

The leftward fluid flow at the ventral surface of the embryonic node is critical for left-right asymmetry determination. This flow is generated by the rotary movement of monocilia. Bending of each cilium was clearly observed during the rotation (Supplementary Movie 13). To investigate the cause of *situs inversus* in *Efcab1*^−/−^ mice, we observed the nodal cilia. Compared with wild type embryos at E7.5, the nodal cilia of *Efcab1*^−/−^ embryos were strikingly sparse or completely absent in some cases and longer microvilli were more prominent on the cell surface (Fig. 4a). The number of motile cilia on the node was less than 15% of the wild-type number (Fig. 4b; Supplementary Movie 14). The fluid flow generated by nodal cilia was clearly leftward in wild-type mice, whereas that in *Efcab1*^−/−^ mice was not detectable or became random in direction (Fig. 4c, d; Supplementary Movie 15 and 16). Fluorescent microbeads at the *Efcab1*^−/−^ node showed a certain but random flow (Fig. 4d) and the velocity was significantly lower than that at the wild-type node (Fig. 4e).

Next, we recorded and analyzed the trajectories of nodal cilia tips. The nodal cilia of wild-type mice showed rotary movement (Fig. 4f); however, *Efcab1*^−/−^ nodal cilia moved in an irregular, not rotary manner and often in planar trajectories (Fig. 4g). To check if this aberrant ciliary motility and fluid flow resulted in *situs inversus*, we examined the gene expression of *Nodal* and *Lefty*, both of which are expressed in the left plate mesoderm (LPM) and are key genes in the determination of left–right asymmetry^35^. Both genes were expressed in the LPM of wild-type E8.0 embryos but in *Efcab1*^−/−^ embryos both genes were expressed on both sides (Fig. 4h).

Since two populations of cilia are known in mouse node, we examined the localization of Efcab1 by immunofluorescent staining. In wild-type embryos, the cilia in the central region of node were recognized with anti-Efcab1 antibody but no significant staining was observed in cilia of the peripheral region (Fig. 4i). As demonstrated by scanning electron microscopy (Fig. 4a, b), we could not detect cilia of the central node region in *Efcab1*^−/−^ mice. However, significant numbers of cilia were observed in the peripheral region of these mice (Fig. 4i).

### Calaxin-deficient zebrafish show *situs inversus* but normal cilia formation in Kupffer’s vesicle

**Fig. 5:**
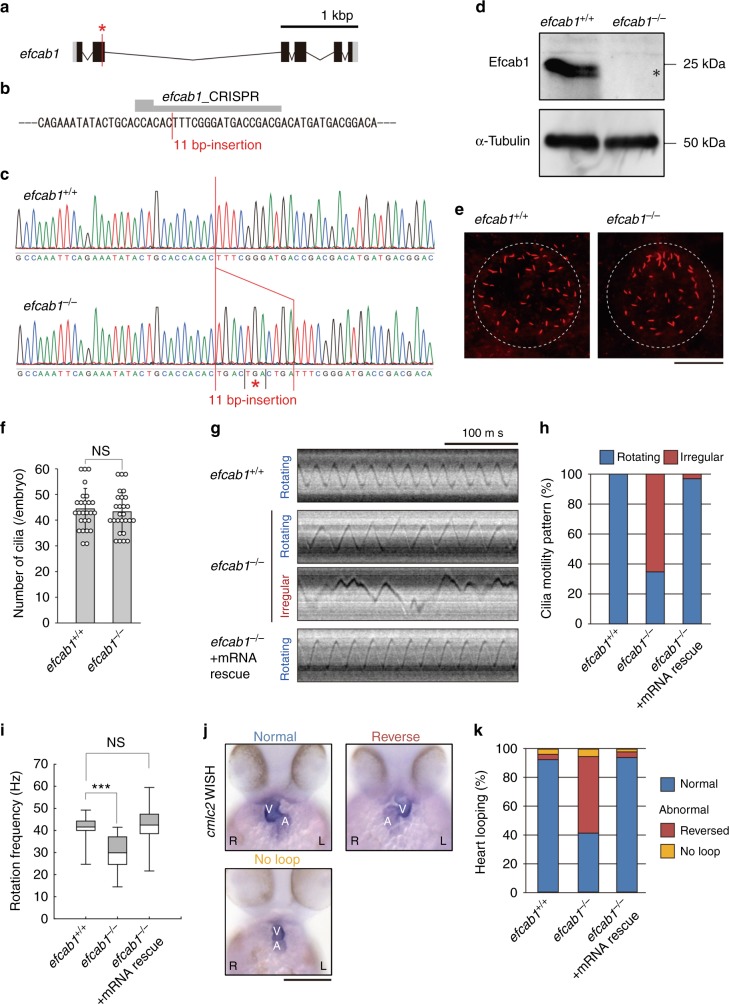
Mutation of zebrafish *efcab1* causes abnormal motility of Kupffer’s vesicle cilia. **a** Genomic organization of zebrafish *efcab1*. Black boxes: exons. Gray boxes: untranslated regions. Red asterisk indicates the genome-editing target site. **b** CRISPR/Cas9 target sequence. **c** Sanger sequencing of *efcab1*^+/+^ and *efcab1*^−/−^ fish around the genome-editing target site. The 11 bp-insertion in *efcab1*^−/−^ includes a stop codon (red asterisk). **d** Immunoblot of testis lysate. The induced mutation deleted Efcab1 (asterisk). α-tubulin: loading control. **e** Kupffer’s vesicle cilia were visualized by immunofluorescence staining with acetylated-tubulin antibodies. **f** Measurement of the number of Kupffer’s vesicle cilia showed no significant differences between *efcab1*^+/+^ and *efcab1*^−/−^ fish. *N* (embryo) = 26 (*efcab1*^+/+^) and 27 (*efcab1*^−/−^). Values indicate mean ± SD. **g** Typical kymographs of Kupffer’s vesicle cilia in *efcab1*^+/+^, *efcab1*^−/−^, and mRNA-rescued *efcab1*^−/−^ fish. Kymograph patterns were categorized into two classes: rotating (blue) and irregular (red). **h** Ratios of each motility class. *N* (cilia) = 55 (*efcab1*^+/+^), 72 (*efcab1*^−/−)^ and 65 (mRNA-rescued *efcab1*^−/−^). **i** Rotational frequencies of Kupffer’s vesicle cilia. Boxes correspond to the first and third quartiles, and whiskers extend to the full range of the data. *N* (cilia) = 55 (*efcab1*^+/+^), 25 (*efcab1*^−/−^) and 63 (mRNA-rescued *efcab1*^−/−^). ***p < 0.001 vs. *efcab1*^+/+^ (Student’s *t*-test). **j** Ventral views of 48 hpf embryos. Heart looping was visualized by whole-mount in situ hybridization of *cmlc2*. L, left; R, right; V, ventricle; A, atrium. **k** Directions of heart looping. *N* (embryo) = 108 (*efcab1*^+/+^), 114 (*efcab1*^−/−^), and 51 (mRNA-rescued *efcab1*^−/−^)

To investigate the conservation of vertebrate calaxin function in the determination of body laterality, we carried out CRISPR/Cas9-mediated knockout of *efcab1* in zebrafish. We identified an 11 bp-insertion in exon 2 of *efcab1*, including an in-frame stop codon (Fig. 5a–c), which resulted in the loss of Efcab1 protein expression (Fig. 5d, Supplementary Fig. 4). In zebrafish, cilia-directed flow in Kupffer’s vesicle (KV) has a critical role in establishing the left-right body axis^36^. In contrast to the drastic loss of nodal cilia formation in *Efcab1*^−/−^ mouse embryos, *efcab1*^−/−^ zebrafish showed normal formation of KV cilia (Fig. 5e); no significant difference was observed in the number of cilia between wild-type and *efcab1*^−/−^ fish (Fig. 5f). However, many of the KV cilia in *efcab1*^−/−^ zebrafish beat with an irregular cycle (Fig. 5g, h) in contrast to the smooth rotary movement in wild-type fish (Supplementary Movie 17). Detailed observation of ciliary movement revealed KV cilia of knockout fish to have abnormal helical movements with less ciliary bending (Supplementary Movie 18). These irregular movements were almost completely rescued by injection of *efcab1* mRNA (Fig. 5g–i; Supplementary Movie 19). The abnormal ciliary movements in *efcab1*^−/−^ fish induced laterality defects in embryos. In wild-type embryos, the heart ventricle loops toward the right and the atrium loops toward the left. However, in almost half of the *efcab1*^−/−^ embryos the direction of the heart loop was reversed. The reversed loop was rescued by injection of *efcab1* mRNA (Fig. 5j, k).

## Discussion

Knockout of mouse calaxin, a Ca^2+^ sensor for ODAs, caused several defects commonly seen in PCD. Despite apparent normal formation of epithelial cilia and sperm flagella, calaxin deficiency resulted in striking inhibition of nodal cilia formation, which could be one reason for the loss of nodal flow. However, induction of left side-specific gene expression requires at least two nodal cilia^37^. Therefore, although *Efcab1*^−/−^ mice have a greatly reduced number of nodal cilia (~5 cilia on average) this cannot explain why half showed *situs inversus*. Rather, we consider aberrant ciliary motility with planar beating, not smooth rotational movement, to be critical for the laterality defect. The common feature of structural normality of 9 + 2 cilia between calaxin knockout mice and PCD patients with DNAH11 (β-type HC of the ODA^24^) mutations suggests a possible relationship between these two proteins.

Calaxin plays an important role in propagation of the asymmetric waveform in ascidian sperm flagella^30^. This is consistent with our results showing suppression of propagation of the pro-hook bend in the sperm flagella of calaxin-deficient mice. However, calaxin-deficient mice are fertile, indicating that calaxin-dependent regulation of the flagellar bend propagation might not be essential for successful fertilization, at least in mice, and that sperm with abnormal propagation of the pro-hook bend can penetrate the zona pellucida to achieve fertilization. This is in contrast to mutant mice lacking sperm-specific calcineurin (PPP3C/PPP3R2), which show defects in bend formation in the proximal region of the flagellum and are defective in zona penetration^38^.

Loss of calaxin resulted in a narrower spatial range of beating in the trachea and brain multicilia of mice. A quite similar effect is also observed in the monocilia of calaxin-deficient sea urchin embryos^32^, suggesting that a major role of calaxin in the regulation of ciliary beating is likely common between mice and sea urchins. In sea urchin embryos, any deficiency of calaxin causes disruption to their basal body orientation, leading to uncoordinated ciliary movement and aberrant swimming^32^. However, despite clear changes in the ciliary waveform and beating of each cilium, the effects of calaxin-knockout on the epithelial fluid flow in mouse multicilia were not overly drastic. It is likely that weak beating of each cilium would not affect the ciliary orientation in multicilia, such as observed in sea urchin monocilia, as it has been suggested that the mechanical feedback of hydrodynamic force within multicilia would compensate for this^39–41^.

Knockout of calaxin caused reduced motility in both the sperm flagella and epithelial multicilia, without any apparent changes in the 9 + 2 structure. In rare cases, ODAs on the doublet 3, 5, and 8 in sperm flagella and the doublet 8 and 9 in trachea cilia were lost, suggesting that calaxin might play some role in the stability of ODAs, particularly for those on the doublets located either side of the plane of central pair complex which are strongly involved for flagellar or ciliary bending^27^. In contrast to the apparently normal formation of sperm flagella and epithelial multicilia, calaxin mutants were deficient in the formation of nodal cilia, indicating a distinct mechanism for ciliary formation in the node.

Why is calaxin essential for the formation of nodal cilia only? It could be related to the type of cilia, i.e., mono-cilia or multi-cilia; however, knockdown of calaxin in sea urchin embryos results in no structural changes in ectoderm monocilia^32^. Moreover, knockout of calaxin in zebrafish resulted in normal formation of KV monocilia. The most probable explanation for the specific effect on ciliary formation is that mouse node cilia have a 9 + 0 axonemal structure, unlike the 9 + 2 structure in other motile cilia. There have been conflicting reports as to whether the dynein species present in nodal cilia possess ODAs and/or IDAs; however, they most probably only possess ODAs^10,42^. When ODAs are assembled in a 9 + 0 arrangement, calaxin might have a critical role in ciliogenesis. This could also indicate a novel mechanism where the Ca^2+^-dependent dynein sensor, calaxin, is involved in intraflagellar transport, docking axonemal components at the basal body/transition zone, or maintenance of axonemal integrity. Although intraflagellar transport is regulated by intracellular Ca^2+^ concentration^43^, the Ca^2+^-binding proteins that respond to the changes in intracellular [Ca^2+^] are yet to be identified. It is possible that Ca^2+^-binding axonemal proteins^3,27,44,45^, such as ODA calaxin, IDA centrin, radial spoke calmodulin (CaM), and CaM-binding proteins in radial spoke/central apparatus, might be involved in the regulation of intraflagellar transport. This idea is consistent with zebrafish 9 + 2 KV cilia with ODAs, IDAs, radial spokes and central apparatus, being normally formed in calaxin-knockout embryos, although motility was sufficiently altered to induce a laterality defect. Two populations of nodal cilia, including those in central region with 9 + 0 and in peripheral region with 9 + 2 structures, have been observed in the mouse node^46,47^. This is consistent with the fact that LRD is only localized in the cilia of central node region^46^ and suggests that calaxin is closely related to the function of LRD motor activity.

Asymmetry of intracellular Ca^2+^ dynamics in the node has been observed^48,49^ but Ca^2+^-dependent regulation of nodal cilia motility is less understood. Attempts to image Ca^2+^ dynamics in the node have produced different conclusions regarding the necessity of Ca^2+^ dynamics in left-right asymmetry^49,50^. No change in motility of KV cilia upon changes in intracellular Ca^2+^ has been observed in zebrafish embryos^51^. However, it is not known how intracellular Ca^2+^ regulates ciliary waveforms. We found that knockout of calaxin causes the change from rotatory movement with proper ciliary bending to planar movement with less bending, indicating that nodal ciliary motility depends on intracellular Ca^2+^-dependent regulation of calaxin. Ca^2+^-binding to calaxin appears to occur at the second EF-hand between concentrations of 10^−7^ to 10^−6^ M intracellular [Ca^2+^], which induces proper propagation of asymmetric flagellar bend in *Ciona* sperm^30,31^. The resting [Ca^2+^] in a mouse node is reported to be ~300 nM^50^, which would represent a threshold for Ca^2+^-binding to calaxin. Therefore, we suggest that Ca^2+^ would be bound to calaxin in the node and would regulate proper ciliary bending, resulting in the generation of left-ward fluid flow.

## Methods

### Generation of *Efcab1*-deficient mice and genotyping

A knockout-first allele system targeting vector for mouse *Efcab1*, the ortholog of the *Ciona* calaxin gene, was obtained from the International Mouse Phenotyping Consortium (https://www.mousephenotype.org/). Chimeric mice were generated using EGR-G101 embryonic stem (ES) cells^52^ by embryo reaggregation of ES cells with ICR-derived 8-cell embryos, and crossed with C57BL/6N female mice to obtain F(1) mice with a conditional knockout allele (tm1a), or crossed with a CAG-Cre transgenic female mouse^53^ to remove the LoxP-flanked region (exon 4) and obtain the line, tm1b. The tm1b line was used to obtain the *Efcab1* knockout line. *Efcab1* tm1a mice were deposited in the Riken BioResource Center with the stock number; RBRC05968 C57BL/6N-Efcab1<tm1a(KOMP)Osb>/19. Animal procedures were performed with the approval of Osaka University, The University of Tsukuba and The National Research Institute for Child Health and Development. Genotyping was subsequently performed by PCR using two pairs of primers from P1 to P3:

Primer 1 (P1) 5′-CAGCTGAGCGCCGGTCGCT-3′

Primer 2 (P2) 5′-TTCCCATCATGGTCATGGTC-3′

Primer 3 (P3) 5′-TCCCAGTACTCCTAGTCACA-3′

### Generation of BAC transgenic mice and rescue experiment

A BAC clone (MSMg01-459G23) containing the full-length MSM mouse *Efcab1* gene was purchased from BACPAC Resources Center (Invitrogen) and microinjected into eggs. For the rescue experiment, transgenic mouse lines expressing *Efcab1* under the control of an *Efcab1* native promoter were produced and transferred onto the *Efcab1*^−/−^ background. Genotyping was performed by PCR using primer pair P4–P5 and subsequent digestion of the PCR product by *SnaB*I:

Primer 4 (P4) 5′-TTCTGCCTTGTGGCTACCTT-3′

Primer 5 (P5) 5′-TCACTGAGCCACACCTGAAG-3′

### Knockout of zebrafish *Efcab1*

Zebrafish embryos and larvae were raised at 28.5 °C in 1/3 Ringer’s solution (39 mM NaCl, 0.97 mM KCl, 1.8 mM CaCl_2_, and 1.7 mM HEPES, pH 7.2). Genome-editing was performed according to a previously reported method^54^ with the target site, CGTCGGTCATCCCGAAAGTG. Animal procedures were performed with the approval of The University of Tokyo.

### Quantitative PCR

Total RNA was extracted from brain, testes or trachea using RNeasy mini kit (Qiagen) according to the manufacturer’s protocols. To remove genomic DNA contamination, extracted RNA was treated with Recombinant DNaseI (Takara). The total RNA was reverse-transcribed into cDNA using Superscript III (Invitrogen). iQ SYBR Green mix (Bio-Rad) was used for PCR reactions carried out with the Thermal Cycler Dice Real Time system (Takara). Primer pairs used for PCR reactions were following:

Q-EFCAB1-Ex4F: 5′-AACAGCCTTCTCAAGCA-3′

Q-EFCAB1-Ex5R: 5′-ACAAAAGACAGCTTCCCA-3′

Q-GAPDH-F: 5′-CATCACTGCCACCCAGAAGACTG-3′

Q-GAPDH-R: 5′-ATGCCAGTGAGCTTCCCGTTCAG-3′

Relative concentrations of Efcab1 mRNA were normalized with GAPDH Ct (cycle threshold) values.

### Whole mount in situ hybridization (WISH)

cDNA clones of *Nodal* and *Lefty* in pBluescript SK were kindly provided by Dr. Hiroshi Hamada (RIKEN, Japan). The latter contained a highly conserved region between closely related *Lefty1* and *Lefty2*. The plasmids were linearized by restriction enzymes and digoxigenin-labeled RNA probes were generated by T7/T3 RNA polymerases. The product was treated with RNA-free DNase and checked by agarose gel electrophoresis. Whole mount *in situ* hybridization was performed according to standard procedures.

For zebrafish, embryos were fixed with 4% paraformaldehyde (PFA) in PBS, and then stored in methanol at −20 °C. After rehydration with PBST, hybridization was performed overnight at 63 °C with digoxigenin-labeled RNA probes. Hybridized specimens were washed with SSC (saline sodium citrate) buffer, then treated with AP-conjugated anti-digoxigenin Fab fragments (1:4000 dilution; Roche) in PBST at 4 °C overnight. After washing with PBST, signals were developed using BM-purple (Roche). When desired intensities of staining were obtained, reactions were stopped. Before observation, specimens were transferred to 80% glycerol/PBS to make them transparent. Images were taken using a stereoscopic microscope (MVX10; Olympus) and a CCD camera (DP73; Olympus).

### In vitro fertilization

Follicular development and ovulation were induced by hCG injection of 8–12-week-old female mice. Mature oocytes were recovered 14–16 h after the hormone treatment. Mature spermatozoa were obtained from the cauda epididymis of 8–10-week-old male mice. Spermatozoa were suspended and pre-incubated in TYH culture medium for 2 h and added to the oocyte suspension at 1.5 × 10^5^ sperm/ml. After incubation at 37 °C for 6 h, male and female pronuclei were stained with Hoechst 33342 and counted under a fluorescence microscope.

### Transmission electron microscopy

Samples were fixed in 2.5% glutaraldehyde, 5 mM MgSO_4_, 0.1 M cacodylate buffer, pH 7.4, for 1 h at room temperature. After washing with 0.1 M cacodylate buffer, pH 7.4, the samples were post-fixed in 1% OsO_4_ on ice for 1 h, dehydrated in a 30–100% EtOH series, substituted by propylene oxide and embedded in Epon 812 or Agar Low Viscosity Resin (LV-Resin). Sections (70 nm) were cut using an ultramicrotome (LEICA, Wetzlar, Germany) and mounted on neoprene-coated grids. Samples were stained with 7% uranyl acetate for 20 min, followed by Reynolds lead staining for 2 min, and observed under a transmission electron microscope (JEM 1200EX, JEOL).

### Scanning electron microscopy (SEM)

Samples were fixed and post-fixed by the same procedure used for transmission electron microscopy, dehydrated in a 30–100% EtOH series, substituted by *t*-butanol and frozen at −30 °C. Samples were then lyophilized in a freeze-drying device (JFD-320, JEOL), mounted on an aluminum block and sputter coated with Au-Pd using an Auto Fine Coater JEC-3000FC (JEOL). Samples were observed under a SEM (JCM5000, JEOL).

### Recording of mouse sperm flagellar motility

Spermatozoa were collected from the cauda epididymis, suspended in TYH culture medium and incubated under mineral oil (Nacalai Tesque) at 37 °C in 5% CO_2_. Sperm motility was observed in a warm chamber (Leja, 20 μm depth, NeuroScience Osceola, WI) with a 10× objective on a BX51 phase contrast microscope (Olympus) and recorded at 500 frames per second (fps) through a HAS-D3 high-speed camera (DITECT, Japan). Velocity of sperm swimming was calculated from the trajectory for 0.5 s.

### Recording of cilia motility in mouse trachea and brain

Tracheal cilia were observed according to a previously described method^55^. Mouse trachea were removed by dissection and placed in DH10 culture medium containing 8.3 g/l DMEM powder (Sigma D5030), 25 mM HEPES-NaOH, pH 7.2, 4.5 g/l glucose, 0.11 g/l sodium pyruvate, 10% FBS. Trachea were opened on the dorsal side and cut into 3 mm squares under a stereoscopic microscope. The tissue pieces were transferred into medium containing 5 mM DTT and incubated for 5 min. The tissue pieces were then transferred to DH10, rinsed for 5 min twice, and observed in a chamber on a glass slide (26 mm × 76 mm, Toshin Riko) with a Scotch tape spacer under a 40× or 100× objective (UPlan FL, Olympus) on a differential interference contrast microscope (BX51, Olympus).

Ependymal cilia were observed according to Ibanez-Tallon et al.^56^. Brains were dissected from 13 to 17-week-old mice, transferred to DH10 culture medium, and trimmed until the epithelium of the lateral ventricle became exposed. Tissue slices of 150 µm thickness were prepared using a Linear Slicer PRO7 (D.S.K Co., Japan) and mounted in medium in a chamber on a glass slide (S1111, Matsunami). The chamber was made by placing a plastic tape as a spacer. The silicon sheet prevented compression of the tissue after sealing the hole with a coverslip. The waveform of ependymal cilia was observed under a 60× objective (UPlan FL, Olympus) on a phase contrast microscope (BX51, Olympus) and recorded at 200 fps through a HAS-220 high-speed camera (DITECT).

### Recording of nodal cilia

Nodal cilia were observed according to Nonaka et al.^57^. Embryos were isolated from decidua by removal of Reichert’s membrane in DH10 medium. Tissue fragments containing the node were excised using a pair of needles and transferred into medium containing 8.3 g/l DMEM powder (Sigma D5030), 25 mM HEPES-NaOH (pH 7.2), 4.5 g/l glucose, 0.11 g/l sodium pyruvate, and 50% rat IC serum^57^. A 0.3 mm thick silicon sheet with a hole was set onto a glass slide and a node fragment was transferred in medium with the nodal cilia face up. The specimen was then covered with a coverslip and observed under a differential interference contrast microscope (BX51, Olympus) with a 40× or 60× objective.

### Analysis of ciliary movement and fluid flow

Fluid flow was traced using 1 µm fluorescent beads (FluoSphere, F-8820, Invitrogen) and recorded at 200 fps by a fluorescence microscope (BX51, Olympus) equipped with a high-speed camera HAS-220 (DITECT) and a 20× objective for tracheal and ependymal cilia or a 60× objective for nodal cilia. Flagellar and ciliary motility and the trajectories of beads were analyzed by motility analysis software, Bohboh^58^. To analyze the trajectories of tracheal ciliary beating, fluorescent beads attached near the tips of cilia were recorded and traced by Bohboh. For nodal cilia, recorded images were processed to reduce the background and the positions of cilium tips were traced by Bohboh.

### Kupffer’s vesicle cilia analysis

Embryos with a Kupffer’s vesicle were selected at 12 hpf and dechrionated before observation. For orientation, embryos were embedded in 0.8% low gelling temperature agarose (Sigma) in 1/3 Ringer’s solution. Motility of Kupffer’s vesicle cilia was observed under bright-field conditions using an inverted microscope (DMI6000B; Leica) and a high-speed camera (HAS-L1; Detect) at 1000 fps.

### Antibodies, immunoblotting, and immunofluorescence microscopy

A cDNA fragment encoding mouse Efcab1 was PCR-amplified, subcloned into a pET-23d vector (Novagen) and expressed in *E*. *coli* BL21 (DE3). The recombinant protein produced was then purified using a His-tag affinity column. A polyclonal antibody against mouse Efcab1 was produced in 16 rabbits via immunization with the recombinant protein. For immunoblot analysis, proteins were extracted by 4 M urea, 1% CHAPS, 20 mM HEPES-NaOH (pH 7.5) or directly by the SDS-buffer for epithelial tissues or sperm, respectively, then separated by SDS-polyacrylamide gel electrophoresis and transferred to a polyvinylidene difluoride membrane. The membranes were blocked by PBST (PBS containing 0.1% Tween 20) containing 7.5% skimmed milk, followed by incubation with the anti-mouse Efcab1 antibody (1:2000) or by a mouse monoclonal antibody against tubulin-α Ab-2 (Clone DM1A) (Thermo Fisher Scientific, 1:10,000). Blots were then incubated with HRP-conjugated secondary antibodies (1:10,000), washed with PBST and developed with ECL-Prime enhanced chemiluminescence substrate kit (GE Healthcares).

Mature spermatozoa were collected from the cauda epididymis, suspended in phosphate-buffered saline (PBS) and immobilized on a poly-lysine coated glass slide. They were fixed in cold methanol (−20 °C), dehydrated with PBS, permeabilized with T-PBS (0.1% Triton X-100 in PBS) and blocked with 10% goat serum in T-PBS for 2 h. After blocking, samples were incubated in the blocking buffer containing a rabbit polyclonal antibody against mouse Efcab1 at 1:100 dilution for 1 h. After washing with T-PBS three times for 1 h, samples were treated with secondary antibodies (Alexa FluorR 488-labeled secondary antibody against rabbit IgG, Invitrogen) at 1:1000 dilution and β-Tubulin-Cy3 (C4585, Sigma-Aldrich) at 1:100 dilution for 1 h. For trachea and brain, tissues were fixed in 4% PFA in PBS for 2 h at 4 °C and then stored in methanol at −20 °C. After rehydration with PBSDT (1% DMSO, 0.1% TritonX-100 in PBS), samples were treated with a blocking buffer containing 10% goat serum in T-PBS. After blocking, samples were incubated in the blocking buffer containing a rabbit polyclonal antibody against mouse calaxin at 1:200 dilution as well as a mouse monoclonal antibody against acetylated α-tubulin (D20G3, Cell Signaling Technology) at 1:400 dilution for 2 h. After washing with T-PBS three times over 1 h, samples were treated with secondary antibodies (Alexa Fluor® 488-labeled secondary antibody against rabbit IgG, Invitrogen, and Alexa Fluor® 546-labeled secondary antibody against mouse IgG, Invitrogen) at 1:200 dilution for 1 h. Samples were washed by T-PBS three times over 1 h, followed by incubation in PBS. For nodal cilia, embryos devoid of Reichert’s membrane were fixed in 4% PFA in PBS for 30 min at 4 °C, washed with T-PBS and treated with cold methanol for 20 min. Subsequent procedures were the same as those employed for trachea and brain, except that antibodies were diluted in the Solution B of Can Get Signal Immunostain (TOYOBO). In trachea and brain samples, 4′,6-diamidino-2-phenylindole (DAPI) was added to PBS at 1 μM before mounting on a glass slide. Observations were made using a fluorescence microscope (Olympus BX53) with a digital camera (DP74, Olympus) for sperm and a confocal microscopy (Fluoview FV10i, Olympus) for epithelial tissues.

For zebrafish, a full-length *efcab1* sequence was subcloned into pGEX-6P-2 (GE Healthcare) and recombinant polypeptides were purified from transformed *Escherichia coli* lysate using Glutathione Sepharose 4B (GE Healthcare). A polyclonal antibody against Efcab1 was raised in guinea pig and an anti-α-tubulin antibody (T9026; Sigma) was purchased for immunoblot analysis. For immunohistochemistry, dechorionated embryos were fixed in 4% paraformaldehyde in PBS, and then stored in methanol at −20 °C. After rehydration with PBSDT, specimens were treated with blocking buffer (1% BSA, 2% normal goat serum in PBSDT). Immunostaining was performed with an anti-acetylated tubulin antibody (1:500 dilution; T6793; Sigma) followed by Alexa Fluor 555 Goat anti-mouse IgG (1:250 dilution; A28180; Thermo). Specimens were mounted with Fluoro-KEEPER Antifade Reagent (Nacalai tesque) and observed using a fluorescence microscope (BX60; Olympus) and a CCD camera (ORCA-R2; Hamamatsu).

### Statistics and reproducibility

Data were collected as follows. For sperm flagella, *N* (sperm) = 299 from four animals (*Efcab1*^+/+^), *N* = 466 from six animals (*Efcab1*^+/−^) and *N* = 465 from six animals (*Efcab*^*1*−/−^). For tracheal flow velocity, values are means ± SE. *N* (beads) = 140 from five animals (*Efcab1*^+/+^), *N* = 101 from three animals (*Efcab1*^+/−^) and *N* = 200 from eight animals (*Efcab1*^−/−^). For brain ependymal cilia, values are means ± SE. *N* (beads) = 221 from three animals (*Efcab1*^+/+^), *N* = 195 from three animals (*Efcab1*^+/−^) and *N* = 155 from five animals (*Efcab1*^−/−^). For counting motile nodal cilia, values are means ± S.E. *N* (embryos) = 18 (*Efcab1*^+/+^) and *N* = 18 (*Efcab1*^−/−^). For nodal flow, values are means ± SE. *N* (beads) = 112 from four embryos (*Efcab1*^+/+^) and *N* = 46 from 10 embryos (*Efcab1*^−/−^). The significance of differences between *Efcab1*^+/+^ and *Efcab1*^−/−^ was calculated using a two-tailed Student’s *t*-test at the significance level *P* < 0.05. All experimental findings were reliably reproduced.

### Data accession

Other relevant information regarding data accession is described in the Supplementary information (Supplementary Note 1).

### Reporting summary

Further information on research design is available in the Nature Research Reporting Summary linked to this article.

## Supplementary information

Supplementary Information

Reporting Summary

Supplementary Movie 1

Supplementary Movie 2

Supplementary Movie 3

Supplementary Movie 4

Supplementary Movie 5

Supplementary Movie 6

Supplementary Movie 7

Supplementary Movie 8

Supplementary Movie 9

Supplementary Movie 10

Supplementary Movie 11

Supplementary Movie 12

Supplementary Movie 13

Supplementary Movie 14

Supplementary Movie 15

Supplementary Movie 16

Supplementary Movie 17

Supplementary Movie 18

Supplementary Movie 19

## Data Availability

Movies associated with the current study are available online at 10.6084/m9.figshare.8121296^59^. All other data generated during and/or analyzed during the currently study are available from the corresponding author on reasonable request.
