# Publisher Correction: Calaxin is required for cilia-driven determination of vertebrate laterality

**DOI:** 10.1038/s42003-019-0512-5

**Published:** 2019-07-04

**Authors:** Keita Sasaki, Kogiku Shiba, Akihiro Nakamura, Natsuko Kawano, Yuhkoh Satouh, Hiroshi Yamaguchi, Motohiro Morikawa, Daisuke Shibata, Ryuji Yanase, Kei Jokura, Mami Nomura, Mami Miyado, Shuji Takada, Hironori Ueno, Shigenori Nonaka, Tadashi Baba, Masahito Ikawa, Masahide Kikkawa, Kenji Miyado, Kazuo Inaba

**Affiliations:** 1 Shimoda Marine Research Center, University of Tsukuba, Shimoda, 415-0025 Japan; 2 Department of Reproductive Biology, National Center for Child Health and Development, Tokyo, 157-8535 Japan; 3 Department of Life Science, School of Agriculture, Meiji University, Kanagawa, 214-8574 Japan; 4 Research Institute for Microbial Diseases, Osaka University, Osaka, 565-0871 Japan; 5 Department of Cell Biology and Anatomy, Graduate School of Medicine, The University of Tokyo, Tokyo, 113-0033 Japan; 6 Department of Molecular Endocrinology, National Research Institute for Child Health and Development, Tokyo, 157-8535 Japan; 7 Department of Systems BioMedicine, National Research Institute for Child Health and Development, Tokyo, 157-8535 Japan; 8 Molecular Function & Life Sciences, Aichi University of Education, Aichi, 448-8542 Japan; 9 Spatiotemporal Regulations Group, Exploratory Research Center on Life and Living Systems (ExCELLS), Okazaki, 444-8585 Japan; 10 Laboratory for Spatiotemporal Regulations, National Institute for Basic Biology, Okazaki, 444-8585 Japan; 11 Faculty of Life and Environmental Sciences, and Life Science Center for Survival Dynamics Tsukuba Advanced Research Alliance (TARA), University of Tsukuba, Tsukuba, 305-8577 Japan

Correction to: *Communications Biology* 10.1038/s42003-019-0462-y, published online 20 June 2019.

In the original published version of the article, panel i was inadvertently omitted from Fig. 4. The figure caption and text were not affected. The error has been corrected in the PDF and HTML versions of the article.

