# Supplementary material for: Calaxin is required for cilia-driven determination of vertebrate laterality
Source: Commun Biol. 2019 Jun 20;2:226. doi: 10.1038/s42003-019-0462-y (PMC6586612; doi:10.1038/s42003-019-0462-y)
Supplement: Supplementary file 1 — Supplementary Information [file 42003_2019_462_MOESM1_ESM.pdf]

Supplementary information

Supplementary Figures

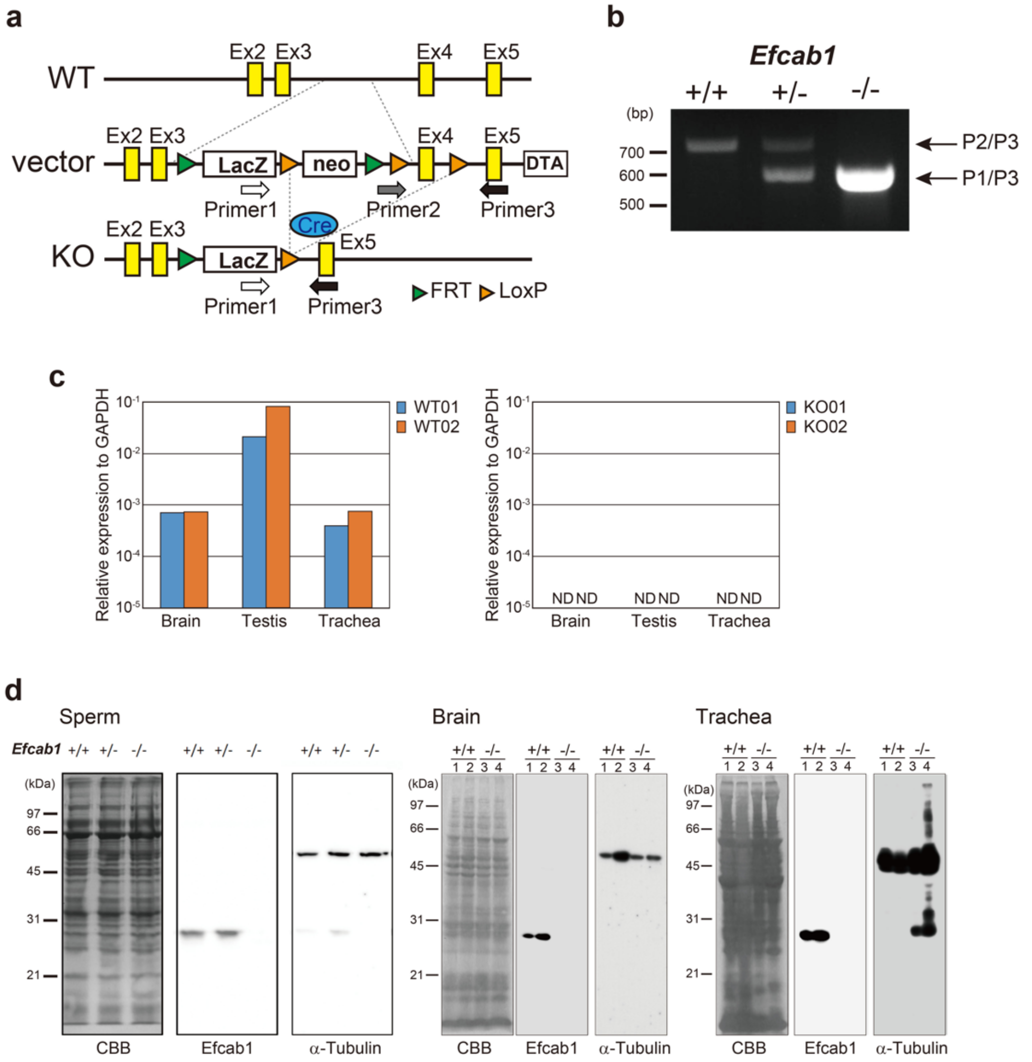

Supplementary Figure 1.

Generation of *Efcab1* mutant mice. (**a**) Schematic depiction of the targeting construct for *Efcab1*. A targeting vector (ID, MGI:1914043) for *Efcab1* containing exon 4 flanked by *loxP* sites. A neomycin resistance cassette (*neo*) and exon 4 were removed from the germline by mating with *CAG-Cre* TG mice. Arrows show primer sets for genotyping. (**b**) Genotyping *Efcab1*$^{+/-}$ and *Efcab1*$^{-/-}$ mice. (c) QPCR data obtained from brain, testis and trachea of two independent batches (KO01 and KO02). *Efcab1* mRNA levels are not detected (ND) in *Efcab1* KO mice. Relative expression against control (GAPDH) is shown as fold differences in the Ct (cycle threshold) values. (**d**) Full length blots of sperm, brain and trachea proteins from *Efcab1*$^{+/+}$, *Efcab1*$^{+/-}$ or *Efcab1*$^{-/-}$ mice probed with an anti-Efcab1 antibody. CBB, protein

staining by Coomassie Brilliant Blue R-250. The blots probed with an anti-α-tubulin antibody represent loading standards.

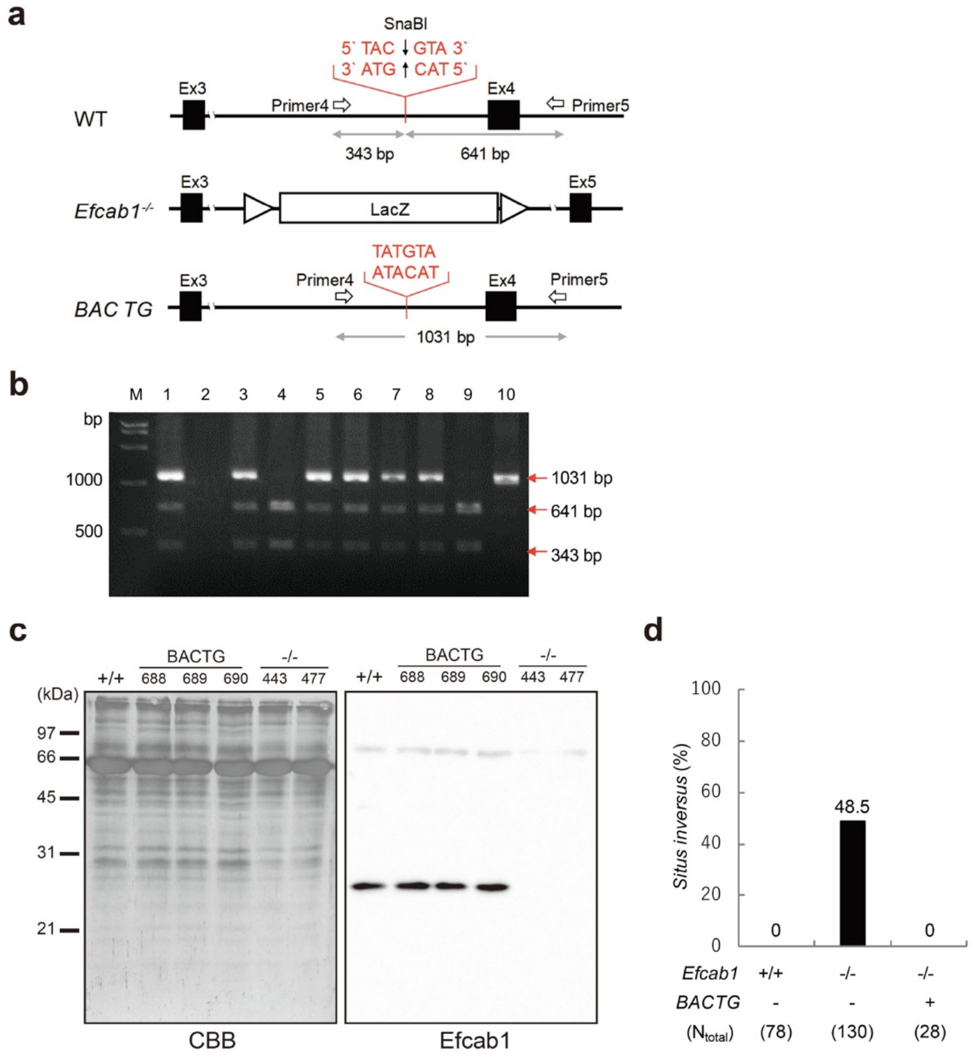

Supplementary Figure 2.

Phenotype rescue in *Efcab1* knockout mice by a bacterial artificial chromosome (BAC) transgene. (**a**) Schematic depiction of the BAC transgene vector. (**b**) Genotyping for both endogenous *Efcab1* and exogenous *Efcab1*. *Efcab1*$^{-/-}$, *BACTG*$^{-}$ (lane 2); *Efcab1*$^{+/-}$, *BACTG*$^{-}$ (lane 4 and 9); *Efcab1*$^{+/-}$, *BACTG*$^{+}$ (lane 1, 3, 5, 6, 7 and 8); *Efcab1*$^{-/-}$, *BACTG*$^{+}$ (lane 10). M, size markers. (**c**) Western blots of whole sperm proteins from wild-type, BAC transgenic rescue, and knockout mice. The numbers above each blot represent numbers of individuals. CBB, protein staining by Coomassie Brilliant Blue R-250. (**d**) Recovery of *situs inversus* in *Efcab1*$^{-/-}$ mice by introducing exogenous *Efcab1*.

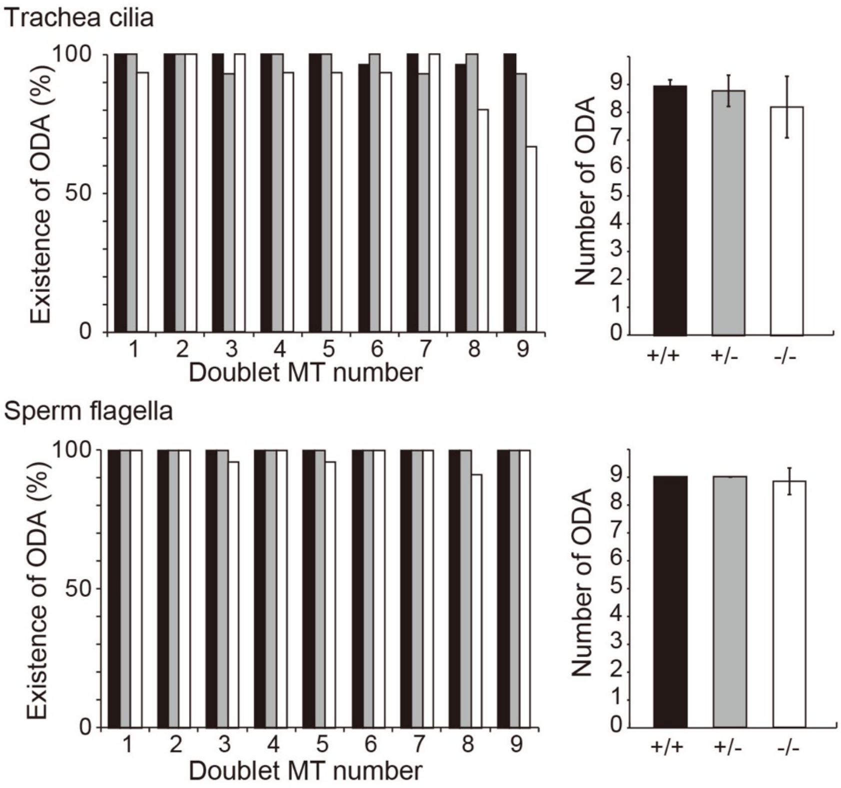

Supplementary Figure 3.

Presence of outer dynein arm in the axoneme of tracheal cilia and sperm flagella in *Efcab1*$^{-/-}$ mice. Presence of the outer dynein arm (ODA) bound to each doublet microtubule (MT) from number 1 to 9 was examined and counted in trachea cilia or sperm flagella obtained from *Efcab1*$^{+/+}$ (black), *Efcab1*$^{+/-}$ (gray) and *Efcab1*$^{-/-}$ (white) mice. Data were collected from thin-section electron microscopy images of axonemes. Numbers of axonemes analyzed in trachea cilia or sperm flagella were N=29 or 16 (*Efcab1*$^{+/+}$), 14 or 18 (*Efcab1*$^{+/-}$), 15 or 23 (*Efcab1*$^{-/-}$), respectively.

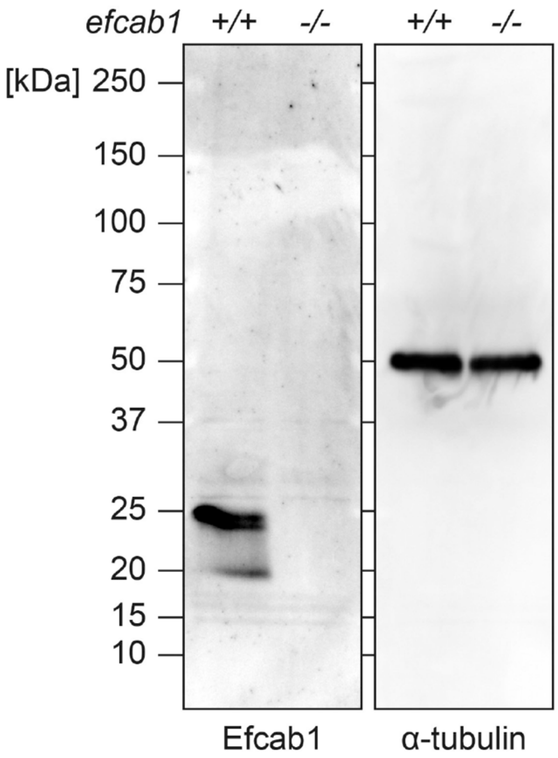

Supplementary Figure 4.

Full length blot of testis proteins from *efcab1*$^{+/+}$ and *efcab1*$^{-/-}$ zebrafish probed with an anti-efcab1 antibody (left). The blot probed with an anti-α-tubulin antibody represents loading standards (right).

Supplementary Table

Table 1.

Genotypes of offspring from heterozygous crosses.

	+/+	+/-	-/-	Total
Male	51	121	29	201
Female	62	124	28	214
Total	113	245	57	415

Data was obtained from 405 offsprings from 74 females.

Supplementary Note

Note 1.

Mouse *Efcab1* reference sequence, NP_080045; Mouse genome information, MGI: 1914043. Relevant information for mouse *Efcab1*, http://www.informatics.jax.org/go/marker/MGI:1914043. BAC clone for rescue of the *Efcab1* deletion, MSMg01-459G23. *Efcab1* tm1a mice stock number, RBRC05968 C57BL/6N-Efcab1<tm1a(KOMP)Osb>/19 (Riken BioResource Center).
