# Supplementary material for: Calaxin is required for cilia-driven determination of vertebrate laterality
Source: Commun Biol. 2019 Jun 20;2:226. doi: 10.1038/s42003-019-0462-y (PMC6586612; doi:10.1038/s42003-019-0462-y)
Supplement: Supplementary file 2 — Reporting Summary [file 42003_2019_462_MOESM2_ESM.pdf]

nature research

Corresponding author(s): [signature]

Last updated by author(s): 2019/04/24

Reporting Summary

Nature Research wishes to improve the reproducibility of the work that we publish. This form provides structure for consistency and transparency in reporting. For further information on Nature Research policies, see Authors & Referees and the Editorial Policy Checklist.

Statistics

For all statistical analyses, confirm that the following items are present in the figure legend, table legend, main text, or Methods section.

n/a	Confirmed	
☐	☒	The exact sample size (n) for each experimental group/condition, given as a discrete number and unit of measurement
☐	☒	A statement on whether measurements were taken from distinct samples or whether the same sample was measured repeatedly
☐	☒	The statistical test(s) used AND whether they are one- or two-sided<br>*Only common tests should be described solely by name; describe more complex techniques in the Methods section.*
☐	☒	A description of all covariates tested
☐	☒	A description of any assumptions or corrections, such as tests of normality and adjustment for multiple comparisons
☐	☒	A full description of the statistical parameters including central tendency (e.g. means) or other basic estimates (e.g. regression coefficient) AND variation (e.g. standard deviation) or associated estimates of uncertainty (e.g. confidence intervals)
☒	☐	For null hypothesis testing, the test statistic (e.g. F, t, r) with confidence intervals, effect sizes, degrees of freedom and P value noted<br>*Give P values as exact values whenever suitable.*
☒	☐	For Bayesian analysis, information on the choice of priors and Markov chain Monte Carlo settings
☒	☐	For hierarchical and complex designs, identification of the appropriate level for tests and full reporting of outcomes
☒	☐	Estimates of effect sizes (e.g. Cohen's d, Pearson's r), indicating how they were calculated

Our web collection on statistics for biologists contains articles on many of the points above.

Software and code

Policy information about availability of computer code

Data collection	Motility analysis software Bohboh (Bohboh soft, Japan).
Data analysis	Motility analysis software Bohboh (Bohboh soft, Japan).

For manuscripts utilizing custom algorithms or software that are central to the research but not yet described in published literature, software must be made available to editors/reviewers. We strongly encourage code deposition in a community repository (e.g. GitHub). See the Nature Research guidelines for submitting code & software for further information.

Data

Policy information about availability of data

All manuscripts must include a data availability statement. This statement should provide the following information, where applicable:

- Accession codes, unique identifiers, or web links for publicly available datasets
- A list of figures that have associated raw data
- A description of any restrictions on data availability

Alldatageneratedoranalyseddduringthisstudyareincludedinthispublishedarticle(anditssupplementaryinformationfiles).

Field-specific reporting

Please select the one below that is the best fit for your research. If you are not sure, read the appropriate sections before making your selection.

☒ Life sciences ☐ Behavioural & social sciences ☐ Ecological, evolutionary & environmental sciences

For a reference copy of the document with all sections, see nature.com/documents/nr-reporting-summary-flat.pdf

Life sciences study design

All studies must disclose on these points even when the disclosure is negative.

Sample size
All the embryos collected and adult tissues (sperm, trachea and brain) randomly selected were used in this study.

Data exclusions
Not applicable.

Replication
We have analyzed from randomly-selected multiple samples and verified by means of appropriate statistical analyses.

Randomization
All the samples were randomly allocated into experimental groups.

Blinding
All the data acquisition and analyses were done under blinding and randomization.

Reporting for specific materials, systems and methods

We require information from authors about some types of materials, experimental systems and methods used in many studies. Here, indicate whether each material, system or method listed is relevant to your study. If you are not sure if a list item applies to your research, read the appropriate section before selecting a response.

Materials & experimental systems

n/a	Involved in the study	
☐	☒	Antibodies
☐	☒	Eukaryotic cell lines
☒	☐	Palaeontology
☐	☒	Animals and other organisms
☒	☐	Human research participants
☒	☐	Clinical data

Methods

n/a	Involved in the study	
☒	☐	ChIP-seq
☒	☐	Flow cytometry
☒	☐	MRI-based neuroimaging

Antibodies

Antibodies used
Anti-mouse EFCAB1 antibody (Abcam, Tokyo; ab121758); Alexa Fluor® 488-labeled anti-rabbit IgG antibody (SIGMA); Cy3-labeled anti-β-tubulin monoclonal antibody (SIGMA); anti-α-tubulin antibody (T9026; Sigma); anti-acetylated tubulin antibody (T6793; Sigma); Alexa Fluor 555 anti-mouse IgG (A28180; Thermo Scientific). Other information on the antibodies and their sources, including laboratory-made antibodies, were described in "Antibodies, immunoblotting and immunofluorescence microscopy" of the "Method" section.

Validation
Specificities of the antibodies used in this study were validated by western blotting, as described in the text.

Eukaryotic cell lines

Policy information about cell lines

Cell line source(s)
EGR-G101 ES cell line was used for generating chimeric mice.

Authentication
We used the cell lines that coauthors have already authenticated (reference 52).

Mycoplasma contamination
All cell lines were tested for mycoplasma contamination.

Commonly misidentified lines
(See ICLAC register)
No commonly misidentified cell lines were used.

Animals and other organisms

Policy information about studies involving animals; ARRIVE guidelines recommended for reporting animal research

Laboratory animals
Mouse (Mus musculus); C57BL/6N and Efcab1 knockout line RBRC05968 C57BL/6N-Efcab1<tm1a(KOMP)Osb>/19 ; males and females; 8-day embryos and adults. Zebrafish (Danio rerio); males and females; embryos and adults. Animal procedures were performed with the approval by the Institutional Animal Care and Use Committee of Osaka University, The University of Tsukuba and The National Research Institute for Child Health and Development for mouse, and with the approval by the Institutional Animal Care and Use Committee of The University of Tokyo for zebrafish.

Wild animals
Not applicable.

Field-collected samples	Not applicable.
Ethics oversight	Not applicable.

Note that full information on the approval of the study protocol must also be provided in the manuscript.
